# Prognostic Value of Preoperative Inflammatory Markers in Melanoma Patients with Brain Metastases

**DOI:** 10.3390/jcm10040634

**Published:** 2021-02-07

**Authors:** Matthias Schneider, Niklas Schäfer, Christian Bode, Valeri Borger, Lars Eichhorn, Frank A. Giordano, Erdem Güresir, Muriel Heimann, Yon-Dschun Ko, Felix Lehmann, Anna-Laura Potthoff, Alexander Radbruch, Christina Schaub, Katjana S. Schwab, Johannes Weller, Hartmut Vatter, Ulrich Herrlinger, Jennifer Landsberg, Patrick Schuss

**Affiliations:** 1 Center of Integrated Oncology (CIO) Bonn, Department of Neurosurgery, University Hospital Bonn, 53127 Bonn, Germany; valeri.borger@ukbonn.de (V.B.); erdem.gueresir@ukbonn.de (E.G.); muriel.heimann@ukbonn.de (M.H.); anna-laura.potthoff@ukbonn.de (A.-L.P.); hartmut.vatter@ukbonn.de (H.V.); patrick.schuss@ukbonn.de (P.S.); 2 Center of Integrated Oncology (CIO) Bonn, Division of Clinical Neuro-Oncology, Department of Neurology, University Hospital Bonn, 53127 Bonn, Germany; niklas.schaefer@ukbonn.de (N.S.); christina.schaub@ukbonn.de (C.S.); johannes.weller@ukbonn.de (J.W.); ulrich.herrlinger@ukbonn.de (U.H.); 3 Department of Anesthesiology and Intensive Care, University Hospital Bonn, 53127 Bonn, Germany; christian.bode@ukbonn.de (C.B.); lars.eichhorn@ukbonn.de (L.E.); felix.lehmann@ukbonn.de (F.L.); 4 Center of Integrated Oncology (CIO) Bonn, Department of Radiation Oncology, University Hospital Bonn, 53127 Bonn, Germany; frank.giordano@ukbonn.de; 5 Center of Integrated Oncology (CIO) Bonn, Department of Oncology and Hematology, Johanniter Hospital Bonn, 53113 Bonn, Germany; yon-dschun.ko@bn.johanniter-kliniken.de; 6 Department of Neuroradiology, University Hospital Bonn, 53127 Bonn, Germany; alexander.radbruch@ukbonn.de; 7 Center of Integrated Oncology (CIO) Bonn, Department of Internal Medicine III, University Hospital Bonn, 53127 Bonn, Germany; katjana.schwab@ukbonn.de; 8 Center of Integrated Oncology (CIO) Bonn, Department of Dermatology and Allergy, University Hospital Bonn, 53127 Bonn, Germany; jenny.landsberg@ukbonn.de

**Keywords:** melanoma, brain metastasis, inflammation, surgical resection, overall survival

## Abstract

Background: Metastatic melanoma disease is accompanied by highly systemic inflammatory responses. The prognostic value of preoperative laboratory inflammation markers in brain metastatic melanoma patients has not been adequately investigated so far. Methods: Preoperative inflammatory blood parameters were correlated to overall survival (OS) rates in melanoma patients that underwent surgery for brain metastasis (BM) between 2013 and 2019 at the authors’ institution. Receiver operating characteristic (ROC) analyses were used for cutoff determination of routine laboratory parameters. Results: Median OS in the present cohort of 30 melanoma patients with surgically treated BM was 7 months (95% confidence interval (CI) 5.7–8.3). Initial elevated C-reactive protein (CRP) levels (>10 mg/L), neutrophil-to-lymphocyte ratio (NLR) ≥ 4, platelet-to-lymphocyte ratio (PLR) ≥ 145, and lymphocyte-to-monocyte ratio (LMR) < 2 were associated with significantly reduced OS rates. Conclusions: The present study identifies several preoperative peripheral inflammatory markers as indicators for poor prognosis in melanoma patients with BM undergoing neurosurgical treatment. Elevated initial CRP values, higher NLR and PLR, and lower LMR were associated with reduced OS and, thus, might be incorporated into preoperative interdisciplinary treatment planning and counseling for affected patients.

## 1. Introduction

The incidence of brain metastases (BMs) is increasing and patients have to face very poor life expectancy [1]. However, the recent approval of immunotherapeutic approaches and immune checkpoint blockade has brought significant advances in the treatment of several cancers that have high rates of BM [2]. For example, immunotherapy has provided crucial advances in the treatment of advanced melanoma [3,4,5,6,7]. Essential in this regard has been a focus on and increasing interest in the inflammatory host response to neoplasms. Thus, there is growing evidence that systemic inflammation may be involved in tumor initiation, progression, and/or metastasis [8]. The systemic response that might be initiated by this process has been attempted to be demonstrated in numerous association studies using a wide variety of laboratory parameters in peripheral blood as potential prognostic indicators in a variety of solid tumors [9,10,11,12,13,14]. Since the treatment of BM is no longer a standalone treatment of a separate department, the neurosurgical removal of BM integrates into an overall interdisciplinary framework [15,16]. Despite all advances in radio- and chemotherapy, surgical resection is often necessary for reasons of cytoreduction, neurological symptoms, and/or space-occupying situations in addition to histological examination.

In this context, it appears that the potential added clinical value of preoperative inflammatory laboratory parameters in the evaluation of melanoma patients who are candidates for resection of brain metastases has not been adequately investigated.

Therefore, the aim of this study was to evaluate overall survival (OS) after cranial surgery in a highly selected cohort of melanoma patients with BM to identify standard preoperative inflammatory values that could help in future surgical case management.

## 2. Materials and Methods

### 2.1. Patients

All melanoma patients with BM who underwent surgical treatment at the authors’ facility between 2013 and 2019 were entered into a computerized database (SPSS, version 25, IBM Corp., Armonk, NY, USA). Institutional ethics committee approval was obtained for this study. Only patients with histopathologically proven BM derived from melanoma were included in further analysis.

Individual treatment decisions were made at the initial presentation of the patient and during follow-up by the weekly institutional interdisciplinary tumor advisory board meetings for the central nervous system, as described previously [17]. After BM resection was recommended by the institutional interdisciplinary tumor board, surgical resection of the BM was performed in all patients analyzed. In the case of multiple brain metastases, the interdisciplinary tumor board had previously decided on the indication for resection of multiple or single lesions (based on size, symptoms, and/or edema). Neurosurgical removal of the metastatic tissue was performed using micro-neurosurgical techniques with standardized patient positioning adapted to the location of the lesion. Furthermore, to ensure patient safety and compliance with standards, the surgical procedures were performed with the aid of neuro-navigation and, if necessary, intraoperative electrophysiological monitoring.

Information, including patient characteristics, radiological features, preoperative laboratory values, preoperative oncological treatment modality, preoperative dexamethasone intake, and functional neurological status at admission, was collected and further analyzed. The Graded Prognostic Assessment (GPA) index was calculated on the basis of the following variables: age, preoperative Karnofsky performance score (KPS), number of CNS metastases, and presence of extracranial metastases, as reported by Spreduto et al. [18]. The Karnofsky performance score (KPS) was used to evaluate patients according to their neurological functional status preoperatively. For further analysis, the results were dichotomized and, thus, a KPS ≥70 was defined as a favorable outcome. Laboratory analysis of C-reactive protein (CRP) and white blood cells (WBC) was performed within 12 h of admission as part of routine laboratory testing. WBC counts (normal range 3.9–10.2 g/L) were divided into two groups, ≤12 g/L and >12 g/L, and CRP (normal range 0–3 mg/L) was dichotomized into ≤10 mg/L and >10 mg/L groups to indicate moderate inflammation, as previously described [16]. Regarding other preoperative inflammatory values, blood was drawn 1 to 14 days before surgery for the detection of lymphocytes (absolute count, 1.1–4 g/L) and absolute neutrophil count (1.5–7.7 g/L). The neutrophil-to-lymphocyte ratio (NLR) was then calculated by dividing the absolute neutrophil count by the absolute lymphocyte count. For the lymphocyte/monocyte ratio (LMR), the absolute lymphocyte count was divided by the absolute monocyte count. Platelet-to-lymphocyte ratio (PLR) was calculated as the absolute platelet count divided by the absolute lymphocyte count. Patients in whom preoperative laboratory values were not available as indicated above were excluded from further analysis.

Overall survival (OS) was measured from the day of BM surgery until death or last observation. Patients in whom no further follow-up information after discharge from in-patient neurosurgical treatment was available due to further offsite treatment were excluded from further analysis. All parameters were compared in terms of OS. 

### 2.2. Statistics

Data analysis was performed using the computer software package SPSS (version 25, IBM Corp., Armonk, NY, USA). Unpaired categorical and binary variables were analyzed in contingency tables using the Fisher exact test. The Mann–Whitney U-test was chosen to compare continuous variables as the data were mostly not normally distributed. The prognostic capacity of NLR, PLR, and LMR was evaluated by the values of the area under the curve (AUC) obtained from the receiver operating characteristic (ROC) curve. OS was analyzed using the Kaplan–Meier survival curves (log-rank test). Results with *p* < 0.05 were considered statistically significant.

## 3. Results

### 3.1. Patient Characteristics

Between 2013 and 2019, a total of 42 patients with melanoma were surgically treated for BM at the authors’ neuro-oncological center. A total of 12 patients were excluded from further analysis after careful review of clinical records due to lack of laboratory values defined as necessary. Therefore, 30 patients with melanoma and surgically treated BM were included in further analysis. The median age was 62 years (range 34–84 years). At admission, patients presented with a median KPS score of 80 (50–100). Median OS for patients with surgically treated BM was 7 months (95% confidence interval (CI) 5.7–8.3). See Table 1 for more patient-specific details.

In seven out of 30 melanoma patients with BM (23%), the diagnosis of the metastatic lesion was also the time of melanoma diagnosis. Within the group of melanoma patients where BM was diagnosed in the course of a known melanoma disease, the median time span between initial melanoma diagnosis and the diagnosis of the BM was 35 months (interquartile range (IQR) 18–65). Patients who had been under chemotherapy in the course of initial melanoma treatment exhibited a median time span of 25 months (IQR 10–50) between initial chemotherapy and resection of BM.

### 3.2. Influence of Preoperative Inflammatory Markers

**Figure 1:**
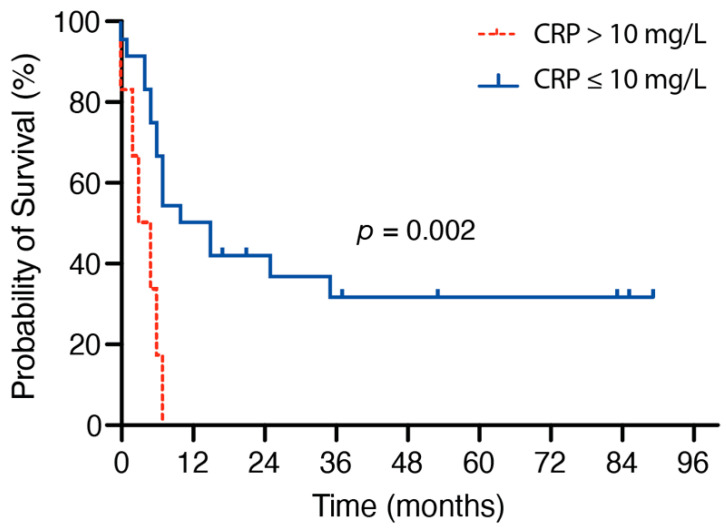
Kaplan–Meier curves for the association of preoperative CRP values and OS. CRP, C-reactive protein; OS, overall survival.

Melanoma patients with an admission CRP ≤ 10 mg/dL achieved a significantly increased OS compared to patients with a preoperative CRP > 10 mg/dL (*p* = 0.002). In detail, the median OS in patients with an admission CRP ≤ 10 mg/L was 10 months (95% CI 2.8–17.2) compared to a median OS of 3 months (95% CI 0.0–6.6) in patients with preoperative CRP > 10 mg/dL (Figure 1). OS rates did not differ significantly between patients with preoperative WBC ≤ 12 g/L and patients with admission WBC > 12 g/L (*p* = 0.5).

Preoperative increased CRP levels did not correlate to smoking anamnesis; one out of six melanoma patients with BM (20%) was a smoker and exhibited preoperative CRP levels > 10 mg/L compared to six smokers out of 24 (25%) patients with preoperative CRP levels ≤ 10 mg/L (*p* = 1.0).

**Figure 2:**
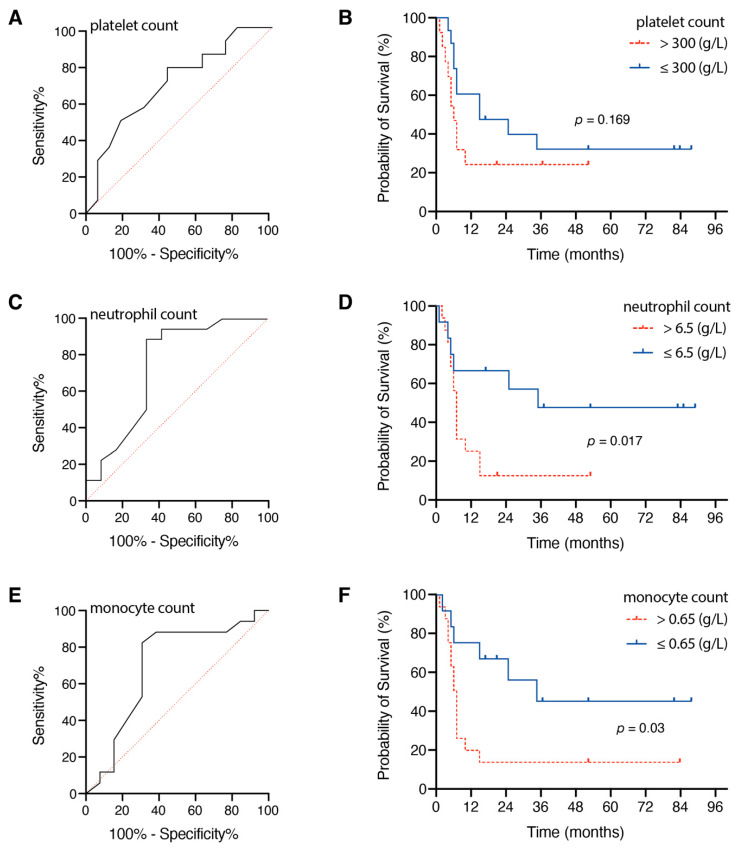
Receiver operating characteristic (ROC) curves (**A**,**C**,**E**) for preoperative blood cell parameters and Kaplan–Meier survival curves (**B**,**D**,**F**) stratified by preoperative blood cell parameters.

Regarding the preoperative platelet count, the optimal cutoff value was 300 g/L, and the AUC was 0.734 (95% CI 0.553–0.915, *p* = 0.033), with a sensitivity of 61% and a specificity of 75% (Figure 2A). Median OS was higher in the group with low initial platelet count (≤300 g/L; 15 months, 95% CI 0.0–30.6) compared to patients with initial high platelet count (>300 g/L; 5 months, 95% CI 2.6–7.4), without reaching a statistically significant difference (*p* = 0.169, Figure 2B).

Concerning the preoperative neutrophil count, the optimal cutoff value was 6.5 g/L, and the AUC was 0.847 (95% CI 0.704–0.990, *p* = 0.001), with a sensitivity of 94% and a specificity of 67% (Figure 2C). Median OS was higher in the group with low initial neutrophil count (≤6.5 g/L; 35 months) compared to patients with preoperative high neutrophil count (>6.5 g/L; 6 months, 95% CI 4.6–7.4), reaching a statistically significant difference (*p* = 0.017, Figure 2D). Preoperative increased WBC levels did not correlate to smoking anamnesis; five out of 17 melanoma patients with BM (29%) with preoperative WBC of >12 g/L were smokers compared to two out of 13 patients (15%) with preoperative WBC ≤ 12 g/L (*p* = 0.4).

In terms of preoperative lymphocytes, no optimal cutoff could be determined in the present dataset (AUC 0.442, 95% CI 0.232–0.652, *p* = 0.597).

The optimal cutoff value with regard to preoperative monocyte count was 0.65 g/L, and the AUC was 0.769 (95% CI 0.596–0.941, *p* = 0.014), with a sensitivity of 78% and a specificity of 75% (Figure 2E). Patients with lower initial monocyte count (≤0.65 g/L; 35 months, 95% CI 6.6–63.4) achieved longer median OS compared to patients with preoperative elevated monocyte count (>0.65 g/L; 6 months, 95% CI 4.7–7.4), reaching a statistically significant difference (*p* = 0.03, Figure 2F).

### 3.3. Influence of Preoperative Inflammatory Marker Ratios

ROC curves were plotted to assess the value of statistically significant values for NLR, LMR, and PLR. AUC was 0.829 for NLR (95% CI 0.679–0.978, *p* = 0.003), 0.88 for LMR (95% CI 0.76–0.999, *p* = 0.001), and 0.718 for PLR (95% CI 0.528–0.907, *p* = 0.047), with the optimal cutoff values at 4 for NLR (72% sensitivity and 83% specificity), 2 for LMR (72% sensitivity and 83% specificity), and 145 for PLR (83% sensitivity and 58% specificity). 

**Figure 3:**
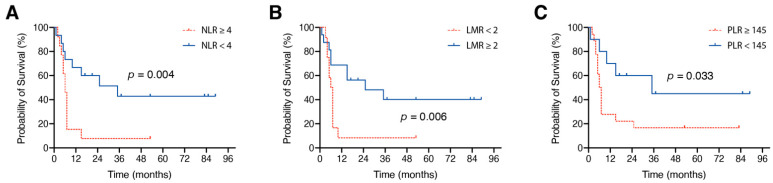
Kaplan–Meier curves for the association of (**A**) NLR, (**B**) LMR and (**C**) PLR and OS. LMR, lymphocyte-to-monocyte ratio; NLR, neutrophil-to-lymphocyte ratio; OS, overall survival; PLR, platelet-to-lymphocyte ratio.

In melanoma patients with BM and an admission NLR < 4, median OS was 35 months, which was significantly different compared to 6 months in patients with an NLR ≥ 4 (*p* = 0.004, Figure 3A). Admission LMR ≥ 2 was significantly associated with longer median OS compared to an admission LMR < 2 (25 months vs. 5 months, *p* = 0.006, Figure 3B). Melanoma patients with a preoperative PLR < 145 achieved significantly longer median OS compared to patients with initial PLR ≥ 145 (35 months vs. 6 months, *p* = 0.033, Figure 3C).

## 4. Discussion

Systemic inflammatory markers in peripheral blood have recently received increasing attention for predicting tumor prognosis among the many potential biomarkers [9,10,11,12,13]. This may be due to the fact that a growing number of patients with advanced cancer are becoming potential candidates for immunotherapy. Here, the initial inflammatory markers or their ratios provide an interesting correlation with the further response to treatment [19]. Furthermore, cancer-associated inflammation both alters and substantially polarizes the tumor microenvironment, and, while not associated with tumor necrosis, it may increase the predisposition toward metastasis [20]. 

In the present study, established peripheral blood laboratory parameters, mostly obtained in routine laboratory tests, and their corresponding ratios were investigated with regard to a potential prognostic significance for the first time among patients with advanced melanoma disease and subsequent brain metastases requiring brain surgery. Strong associations were found between preoperative NLR < 4, LMR ≥ 2, and PLR < 145 and a prolonged overall survival. Furthermore, patients with a CRP > 10 mg/L at the time of preoperative admission showed significantly inferior overall survival.

The levels of C-reactive protein, a prototypical acute-phase reactant, increase in inflammatory conditions [21]. Chronic inflammation and high CRP levels are associated with poor survival of several types of cancer, including renal cell, lung, pancreatic, and breast cancer [21]. With regard to patients with melanoma, recent data have suggested that CRP might bind to T cells and, thus, suppress their function in the earliest stages of T-cell activation in a dose-dependent manner [21]. This could provide CRP with a direct immunosuppressive role that might explain the poor survival in melanoma patients with high CRP levels. The outcomes of the present study underscore this aspect pertaining to melanoma patients with brain metastasis requiring surgery, as a preoperative CRP value of >10 mg/L was shown to be significantly associated with poorer overall survival. Several previous studies reported findings linking elevated preoperative CRP levels to increased lymph node metastasis and limited OS in breast cancer patients [22,23,24]. CRP levels have been reported to be significantly more increased in metastatic than nonmetastatic cancer patients, highlighting a particular importance of CRP as a systemic metabolite in advanced metastatic cancer disease [24]. Similarly, in Non-Small Cell Lung Cancer patients with CRP levels higher than 40 mg/L were more likely to have a metastatic disease with a specificity of 100% [25]. Considering the existing literature, CRP appears to constitute biomarker of growing importance for metastatic stages and survival in cancer patients. However, the very nonspecific nature of elevated CRP must not be neglected [26]. The occurrence of increased adverse cardiovascular complications in the further course of treatment of these patients represents another possibility, as elevated CRP also seems to be associated with such a risk [27].

With regard to more specific inflammatory markers, white blood cell counts (i.e., neutrophils, lymphocytes, platelets, and monocytes) and their ratios to each other (e.g., NLR, PLR, and MRL) are increasingly emphasized because hematologic testing is routinely performed in cancer patients in clinical practice and, biologically, activation of systemic inflammation is accompanied by changes in circulating white blood cells, such as the appearance of neutrophilia with associated lymphocytopenia [28,29]. Platelets, in turn, contribute to the perpetuation of proliferative signs, along with cancer cells that produce platelet-derived growth factors in abundant amounts. These growth factors may promote tumor progression [30]. High platelet counts in peripheral blood have been associated with poorer prognosis in patients with various cancer diagnoses [31]. This is consistent with available data on patients with advanced melanoma and brain metastases requiring surgery. Our data also support the perception that relative lympho-cytopenia might be associated with poor prognosis in cancer patients [32]. In our study, the patient group with poorer outcomes (high NLR, high PLR, and low MRL) had significantly lower peripheral blood lymphocyte counts in subsequent analyses. These findings are in line with a recent meta-analysis which showed a strong correlation between elevated PLR and worse OS in pancreatic cancer, renal cell carcinoma, and mesothelioma, among others, thus suggesting PLR to constitute an independent factor associated with restricted survival outcome [31]. Similarly to our findings in brain metastasizing melanoma disease, elevated NLR was found to correlate to poor survival outcomes in several cancer entities [33]. 

For melanoma, previous studies suggested that preoperative peripheral blood inflammatory markers correlate with prognosis in patients with melanoma at any stage [29,34,35,36]. Nevertheless, the results of the present study concurred with previously reported findings in the literature only in part, because so far, no prognostic analysis has been performed in advanced melanoma with metastasis to the brain and its need for surgery.

### Limitations

Although our data and results were thoroughly honed and calculated, this study had some limitations. Given the highly selective patient cohort of brain metastatic melanoma patients, only 30 patients were examined in this study, which may yield inconsistent results due to the small sample size. Furthermore, data collection was performed retrospectively and patients were not randomized, but treated according to the preferences of the treating physicians. According to the so-called “one in 10 rule”, only one predictive value should be studied for every 10 events, i.e., for logistic regression analysis, the number of events is given by the smallest of the outcome categories. With regard to the low number of highly selected patients with brain metastasizing melanoma disease and neurosurgical intervention, additional multivariate analysis was not applicable. Nevertheless, this study provides a basis for future research by predicting prognosis in melanoma patients with BM using circulating inflammatory markers and validating a specific threshold for each marker.

## 5. Conclusions

In the present study, preoperative peripheral inflammatory markers (NLR, LMR, and PLR) were identified as indicators of prognosis in melanoma patients with BM undergoing neurosurgical treatment. Higher initial NLR and PLR and lower LMR were associated with poorer OS and, thus, might be incorporated into preoperative interdisciplinary treatment planning/counseling for affected patients.

## Figures and Tables

**Table 1:** Patient characteristics.

	Patients with Melanoma and BM (*n* = 30)
Median age at surgery (years)	62 (43–84 **^1^**)
Female sex	11 (37%)
Median GPA index	2.5 (0.5–4.0)
Median preoperative KPS	80 (50–100)
Previous oncological therapy ^2^	12 (40%)
Preoperative corticosteroid intake	13 (43%)
Multiple BM	4 (13%)
Admission median CRP (mg/L)	12.3 (0.2–102.8)
Admission median WBC (g/L)	12.9 (4.3–32.2)
Admission median neutrophils (g/L)	8.4 (2.04–30.02)
Admission median lymphocytes (g/L)	1.7 (0.25–3.26)
Admission median monocytes (g/L)	0.79 (0.2–1.83)
Admission median platelets (g/L)	306.8 (179–603)
Median OS (months)	7 (95% CI 5.7–8.3)

^1^ Range. ^2^ Chemo- and/or immunotherapy. BM, brain metastasis; CRP, C-reactive protein; KPS, Karnofsky performance status; WBC, white blood cells; GPA, Graded Prognostic Assessment; OS, overall survival; CI, confidence interval. Laboratory values are described as median and range.
